# Cardiac autonomic dysfunction in adult congenital heart disease

**DOI:** 10.1186/s12872-023-03558-4

**Published:** 2023-10-21

**Authors:** Carmen Pizarro, Franziska Luise Bosse, Charlotte Begrich, Barbora Reznakova, Thomas Beiert, Jan Wilko Schrickel, Georg Nickenig, Dirk Skowasch, Diana Momcilovic

**Affiliations:** University Hospital Bonn, Venusberg-Campus 1, 53127 Bonn, Germany

**Keywords:** Adult congenital heart disease, Heart rate variability, Holter monitoring, Time domain analysis, Frequency domain analysis, Prognosis

## Abstract

**Purpose:**

Due to recent advances in diagnosis and treatment, the number of adults with congenital heart disease (ACHD) has substantially increased. This achievement is mitigated by rhythm disorders. Here, we sought to determine alterations in heart rate variability (HRV) and their prognostic value in ACHD.

**Methods:**

Ninety seven ACHD patients (39.2 ± 14.1 years, 51.5% female) and 19 controls (39.7 ± 15.0 years, 47.4% female) underwent 24-h Holter monitoring.

**Results:**

As compared to controls, ACHD patients offered a significantly higher burden of premature ventricular contractions (*p* = 0.02) and decreased HRV indices (natural logarithmic transformation of very low frequency (lnVLF): 7.46 ± 0.76 ms^2^ vs. 7.91 ± 0.92ms^2^, *p* = 0.03; natural logarithmic transformation of low frequency (lnLF): 6.39 ± 0.95ms^2^ vs. 7.01 ± 1.07ms^2^, *p* = 0.01; natural logarithmic transformation of the ratio of low to high frequency spectra (lnLF/HF): 0.81 ± 0.74 vs. 1.17 ± 0.51, *p* = 0.04). No differences in HRV measures were observed across ACHD lesion groups. NT-proBNP levels were significantly related to both time and frequency domain indices (natural logarithmic transformation of the standard deviation of NN intervals (lnSDNN): Spearman´s rho = -0.32, *p* = 0.001; natural logarithmic transformation of the standard deviation of the average NN intervals for each 5-min segment of a 24-h Holter monitoring (lnSDANN): Spearman´s rho: -0.33, *p* = 0.001; natural logarithmic transformation of the total power (lnTP): Spearman´s rho: -0.25, *p* = 0.01; lnVLF: Spearman´s rho: -0.33, *p* = 0.001; lnLF: Spearman´s rho: -0.35, *p* < 0.001; lnLF/HF: Spearman´s rho: -0.34, *p* = 0.001).

After a mean follow-up of 3.9 ± 0.7 years, 8 patients died and 3 patients survived sudden cardiac death (SCD). Several HRV parameters were significantly higher in event-free ACHD patients than in those who died or survived SCD (natural logarithmic transformation of the average of the standard deviations of NN intervals for each 5-min segment of a 24-h Holter monitoring (lnASDNN): *p* = 0.04; lnPNN30: *p* = 0.04; lnVFL: *p* = 0.03; lnLF: p < 0.01). On univariate Cox regression analysis, the time domain indices lnSDNN, lnASDNN and lnPNN30, as well as the frequency domain parameters lnTP, lnVLF and lnLF were associated with death and survived cardiac arrest.

**Conclusion:**

ACHD is accompanied by HRV impairment that carries prognostic implications on ACHD mortality and survived SCD.

## Introduction

Congenital heart disease with an approximate prevalence of 9 per 1000 new-borns worldwide is a common inborn defect which used to carry a very poor prognosis. However, due to advancements in diagnosis and management, > 90% of these patients reach adulthood nowadays [1]. This has led to a growing population of adults with congenital heart disease (ACHD). Despite medical advances, ACHD patients are afflicted by long-term complications including arrhythmias. They encompass the whole range of atrial and junctional tachycardias, bradyarrhythmias and ventricular arrhythmias which are a major cause of hospitalization and mortality [2]. In the ACHD population, sudden cardiac death (SCD) constitutes up to 40% of all deaths. In keeping with this, management of arrhythmias is an essential compound of ACHD care.

The autonomic nervous system plays an important role in the modulation of cardiac electrophysiology and arrhythmogenesis. Cardiac autonomic nervous dysfunction is associated with the occurrence of malignant arrhythmias and an increased risk of death in acquired cardiac diseases. Autonomic dysfunction is also common in patients with ACHD [3, 4] and small studies already suggested that it could be a means of risk assessment in this population [5]. Measurement of heart rate variability (HRV) represents a non-invasive approach to evaluate alterations in cardiac autonomic function [6]. HRV describes the fluctuations of normal heart beat intervals during electrocardiography (ECG)-monitoring. Decreased HRV is a strong predictor of adverse prognosis. HRV reduction has been shown to have prognostic implications in coronary heart disease, especially in the early post-myocardial infarction period [7]. In acquired heart failure, reduced HRV has been identified as an independent predictor of ventricular arrhythmias, SCD and mortality [8]. However, in the population of ACHD patients, its diagnostic and prognostic value remains less well defined.

Thus, the aim of this prospective cohort study was i) to identify alterations in HRV in ACHD patients as a function of the underlying congenital lesion, ii) to compare the results with those obtained in healthy controls and iii) to assess the prognostic value of HRV measurements for risk stratification in ACHD patients.

## Material and methods

### Study population

Between January 2018 and January 2020, ACHD patients aged ≥ 18 years were screened for this prospective cohort trial. All patients received treatment at the Department of Cardiology, Hospital of Bonn (Bonn, Germany). Patients with an implanted cardiac pacemaker were excluded from study participation. In line with the current guidelines on ACHD management [9], patients were classified into four lesion groups namely a) shunt lesions, b) left-sided obstructive lesions, c) right-sided lesions and d) complex lesions.

Healthy controls without preexisting structural cardiac disease were recruited by screening invitation from the general population and were matched for age and gender. Both ACHD patients and controls underwent a standardized questionnaire-based clinical evaluation, 24-h Holter monitoring, transthoracic echocardiography and laboratory testing. Written informed consent was obtained from each participant. The study was performed in line with the principles of the 1975 Declaration of Helsinki. Approval was obtained from the Medical Ethics Committee of the University of Bonn (Germany).

The study´s primary objective was to analyse the frequency and impact of altered HRV in ACHD patients. Its primary outcome was the composite of death or survived cardiac arrest during follow-up in ACHD.

### Holter monitoring

Holter monitoring was carried out on a five lead 24-h ECG (SpiderView, MicroPort CRM, Paris, France). Holter recordings were manually analysed by a cardiologist who discarded artefacts and edited misclassified heartbeats. All ECG data were examined for presence of arrhythmic events comprising premature atrial and ventricular contractions (PAC and PVC, respectively), as well as supraventricular and ventricular salvos and tachyarrhythmias.

HRV analyses were performed by use of SyneScope Version 3.10 software (MicroPort CRM, Paris, France) and comprehended both time and frequency domain measures. For time domain analysis, normal-to-normal (NN) sinus interbeat intervals were evaluated, resulting in the following measures: 1.) standard deviation of NN intervals (SDNN); 2.) standard deviation of the average NN intervals for each 5-min segment of a 24-h ECG recording (SDANN); 3.) average of the standard deviations of NN intervals for each 5-min segment of a 24-h ECG recording (ASDNN); 4.) root mean square of differences between NN intervals (RMSSD); 5.) percentage of NN intervals that differ from the prior interval by at least 30 ms or 50 ms (PNN30 and PNN50, respectively). Frequency domain HRV analysis was conducted by spectral analysis based on fast Fourier transform. The following frequency bands were obtained: 1.) very low frequency (VLF) spectrum (0.0033–0.04 Hz) that is generated by the stimulation of afferent sensory neurons in the heart and modulated by physical activity and stress response; 2.) low frequency (LF) spectrum (0.04–0.15 Hz) that reflects both vagal and sympathetic influences; 3.) high frequency (HF) spectrum (0.15–0.40 Hz) that represents parasympathetic activity; 4.) the ratio of low to high frequency spectra (LF/HF ratio) that estimates the ratio between sympathetic to parasympathetic nervous system activity; 5.) total power (TP) spectrum (≤ 0.40 Hz) that sums up the aforementioned spectra and captures the total variance in HRV. Due to the skewed, not normal distribution of time and frequency domain variables, a natural logarithmic transformation (ln) was applied for all HRV measurements.

### Transthoracic echocardiography

A complete transthoracic echocardiographic study was performed by experienced cardiac sonographers trained in the field of ACHD according to current guidelines [10]. Ultrasound data were acquired with a 2.5 MHz phased-array transducer using a commercially available ultrasound system (iE33, Philips Medical Systems, Andover, Massachusetts; GE Vivid E9, GE Health Medical, Horten, Norway). Systolic ventricular function was assessed by biplane Simpson´s method from volumes obtained by the summation of a stack of elliptical discs. In case of a morphologic right systemic ventricle, ventricular function was quantified by cardiac magnetic resonance.

### Laboratory testing

The study participants underwent blood sampling. Measurements encompassed a complete blood cell count, N-terminal prohormone of brain natriuretic peptide (NT-proBNP), serum creatinine and thyroid stimulating hormone (TSH) levels.

### Statistical analysis

Continuous variables are presented as mean ± standard deviation, if normally distributed, or as median and interquartile range (IQR, quartile 1/quartile 3), if not normally distributed. HRV indices underwent logarithmic transformation. Continuous variables were tested for having a normal distribution with the use of the Kolmogorov–Smirnov test. Categorical variables are given as absolute numbers and percentages. Comparison across the four ACHD lesion groups of continuous variables was carried out by univariate ANOVA or Kruskal–Wallis *H* test (if normality assumption was violated). Differences in categorical variables were analysed by Fisher´s exact test. Comparison between the total patient cohort and controls was performed by Student´s *t*-test or Mann–Whitney *U* test (if normality assumption was violated). If the global test was significant, Bonferroni correction was applied for post hoc analysis. Spearman’s correlation coefficients were used to establish associations. To assess the relationship between variables and mortality, a univariate Cox proportional hazard regression analysis was performed. Multivariate Cox regression analysis was intended in case of a sufficient number of events per variable. Statistical significance was assumed when the null hypothesis could be rejected at *p* < 0.05. Statistical analyses were conducted with SPSS Statistics version 26.0 (IBM, Armonk, NY, USA).

## Results

### Clinical characteristics

A total of 102 ACHD patients were screened for this prospective cohort trial. Five patients were excluded due to incomplete Holter monitoring data, resulting in a final ACHD study population size of 97 participants.

Table 1 summarizes the demographic and clinical features of study participants. Overall, patients were middle-aged (39.2 ± 14.1 years); the gender ratio was largely balanced (51.5% female). As to New York Heart Association (NYHA) functional class, the vast majority was asymptomatic or oligosymptomatic. The systolic function of the systemic ventricle was predominantly preserved with a mean ejection fraction (EF) of 58.3 ± 8.7%. 33 out of 97 patients (34.0%) had a history of arrhythmias, mainly supraventricular in nature; only two patients had a history of non-sustained ventricular tachycardia. 10.3% of ACHD patients were under antiarrhythmic medication. As aforesaid, ACHD patients were categorized into four lesion groups according to their haemodynamics. 26/97 patients (26.8%) had shunt lesions, 21/97 patients (21.6%) presented left-sided obstructive lesions, 28/97 patients (28.9%) offered right-sided lesions; the remaining 22/97 patients (22.6%) had complex lesions. An overview of the underlying defects and prior corrective repair is given in Table 2. ACHD patients were compared to 19 controls without preexisting structural cardiac disease, though three controls were on beta-blockers due to arterial hypertension. When ACHD patients were compared to controls, patients offered a significantly impaired EF (58.3 ± 8.7% vs. 64.0 ± 4.2%; *p* < 0.01) and higher NT-proBNP levels (140 pg/ml (IQR 60–333) vs. 47 pg/ml (IQR 33–82); *p* < 0.001). Likewise, after introduction of established NT-proBNP cut-offs at 125 pg/ml and 300 pg/ml, significant differences between ACHD patients and controls could be upheld [11].

**Table 1:** Baseline characteristics of the study population

	All patients (*n* = 97)	Shunt lesions (*n* = 26)	Left-sided obstructive lesions (*n* = 21)	Right-sided lesions (*n* = 28)	Complex lesions (*n* = 22)	Controls (*n* = 19)	*p*-value*	*p*-value**
Demographics								
Female	50 (51.5%)	16 (61.5%)	10 (47.6%)	13 (46.4%)	11 (50.0%)	9 (47.4%)	0.81^F^	0.70^F^
Age [years]	39.2 ± 14.1	43.1 ± 13.8	37.3 ± 13.7	39.7 ± 15.0	35.6 ± 13.1	39.7 ± 15.0	0.88^ T^	0.28^A^
BMI [kg/m^2^]	25.9 ± 5.9	26.7 ± 6.3	25.9 ± 6.3	25.0 ± 4.4	26.1 ± 6.9	23.7 ± 4.7	0.13^ T^	0.77^A^
Previous reparative intervention	77 (79.4%)	17 (65.4%)	19 (90.5%)	26 (92.9%)	15 (68.2%)	0 (0%)	< 0.001^F^	0.02^F^
NYHA functional class							< 0.001^F^	0.66^F^
I	52 (53.6%)	15 (57.7%)	14 (66.7%)	13 (46.4%)	10 (45.5%)			
II	38 (39.2%)	10 (38.5%)	7 (33.3%)	12 (42.9%)	9 (40.9%)			
III	6 (6.2%)	1 (3.8%)	0 (0%)	2 (7.1%)	3 (13.6%)			
IV	1 (1.0%)	0 (0%)	0 (0%)	1 (3.6%)	0 (0%)			
Biochemistry								
NT-proBNP [pg/ml]	140 (62 – 333)	164 (52–297)	104 (38–311)	171 (93–413)	178 (66–421)	47 (33–82)	< 0.001^ M^	0.35^ K^
NT-proBNP ≥ 125 [pg/ml]	48 (49.5%)	12 (46.2%)	7 (33.3%)	17 (60.7%)	12 (54.5%)	3 (15.8%)	0.01^F^	0.39^F^
NT-proBNP ≥ 300 [pg/ml]	28 (28.9%)	5 (19.2%)	6 (28.6%)	10 (35.7%)	7 (31.8%)	0 (0%)	0.003^F^	0.72^F^
Serum creatinine [mg/dl]	0.76 ± 0.20	0.97 ± 0.21	0.77 ± 0.14	0.75 ± 0.21	0.80 ± 0.22	0.77 ± 0.13	0.84^ T^	0.57^A^
TSH [mU/l]	2.12 ± 1.25	1.87 ± 0.81	1.94 ± 1.2	2.69 ± 1.66	1.97 ± 1.07	1.63 ± 1.03	0.12^ T^	0.09^A^
Systemic ventricle EF [%]	58.3 ± 8.7	59.0 ± 5.1	60.3 ± 7.9	58.3 ± 9.5	54.6 ± 11.9	64.0 ± 4.2	< 0.01^ T^	0.052^A^
Medication use								
Beta-Blocker	41 (42.3%)	10 (38.5%)	12 (57.1%)	10 (35.7%)	9 (40.9%)	3 (15.8%)	0.04^F^	0.47^F^
Antiarrhythmics	10 (10.3%)	2 (7.7%)	0 (0%)	5 (17.9%)	3 (13.6%)	0 (0%)	0.36^F^	0.20^F^

Data are presented as total number (percentage), mean ± standard deviation or median (interquartile range). * *p*-values refer to data comparison between the total patient group and controls (^F^ = Fisher´s exact test, ^M^ = Mann–Whitney *U* test, ^T^ = unpaired *t*-test). ** *p*-values refer to data comparison between all four ACHD subgroups (^A^ = univariate ANOVA, ^K^ = Kruskal–Wallis test, ^F^ = Fisher´s exact test). *Abbreviations*: *EF* ejection fraction, *NT-proBNP* N-terminal prohormone of brain natriuretic peptide, *NYHA* New York Heart Association, *TSH* thyroid stimulating hormone

**Table 2:** Overview of specific underlying defects and previous corrective repair

Underlying cardiac defect	Total	Previous corrective repair	No previous corrective repair
**Shunt lesions (*n* = 26)**			
Ventricular septal defect	10 (10.3%)	7 (7.2%)	3 (3.1%)
Atrial septal defect	6 (6.2%)	3 (3.1%)	3 (3.1%)
Atrioventricular septal defect	5 (5.2%)	4 (4.1%)	1 (1.0%)
Anomalous pulmonary venous connection	3 (3.1%)	2 (2.1%)	0 (0%)
Patent ductus arteriosus	2 (2.1%)	1 (1.0%)	1 (1.0%)
**Left-sided obstructive lesions (*n* = 21)**			
Congenital valvular aortic stenosis	10 (10.3%)	9 (9.3%)	1 (1.0%)
Coarctation of aorta	9 (9.3%)	9 (9.3%)	0 (0%)
Subaortic stenosis	1 (1.0%)	1 (1.0%)	0 (0%)
Congenital mitral stenosis	1 (1.0%)	0 (0%)	1 (1.0%)
**Right-sided lesions (*n* = 28)**			
Tetralogy of Fallot	17 (17.5%)	16 (16.5%)	1 (1.0%)
Pulmonary stenosis	7 (7.2%)	7 (7.2%)	0 (0%)
Ebstein anomaly	4 (4.1%)	3 (3.1%)	1 (1.0%)
**Complex lesions (*n* = 22)**			
Transposition of the great arteries	8 (8.2%)	7 (7.2%)	1 (1.0%)^a^
Fontan palliation	5 (5.2%)	5 (5.2%)	0 (0%)
Eisenmenger syndrome	5 (5.2%)	1 (1.0%)	4 (4.1%)
Heterotaxy syndrome	2 (2.1%)	1 (1.0%)	1 (1.0%)
Truncus arteriosus	1 (1.0%)	1 (1.0%)	0 (0%)
Coronary artery fistula	1 (1.0%)	0 (0%)	1 (10%)

Data are presented as total number and percentage of the entire patient population (in parentheses)

^a^Congenitally corrected transposition of the great arteries

### Holter monitoring

Arrhythmic events over 24 h are displayed in Table 3. The number of study participants that presented PVCs was significantly higher in the patient group than amongst controls (77.3% vs. 52.6%, *p* = 0.02). However, there were no differences between both groups in terms of PAC, supraventricular or ventricular tachyarrhythmias. When arrhythmic events were compared over ACHD patient groups, only PVC counts varied significantly. Bonferroni adjustment ascribed this effect to differences between the right-sided lesion group, on the one hand, and the shunt and left-sided obstructive lesion group, on the other hand (*p* = 0.004 and *p* = 0.04, respectively).

**Table 3:** Arrhythmic events during Holter monitoring

	All patients (*n* = 97)	Shunt lesions (*n* = 26)	Left-sided obstructive lesions (*n* = 21)	Right-sided lesions (*n* = 28)	Complex lesions (*n* = 22)	Controls (*n* = 19)	*p*-value*	*p*-value**
PAC								
Number of participants	81 (83.5%)	21 (80.8%)	17 (81.0%)	23 (82.1%)	20 (90.9%)	18 (94.7%)	0.69^F^	0.97^F^
Events/24 h	29 (10–285)	20 (6–116)	25 (10–365)	37 (15–901)	35 (10–322)	12.5 (9–27)	0.16^ M^	0.46^ K^
PAC salvos								
Number of participants	51 (52.6%)	12 (46.2)	14 (66.7%)	14 (50.0%)	11 (50.0%)	11 (57.9%)	1.00^F^	0.53^F^
Events/24 h	6 (2–15)	2 (1–83)	4 (2–7.5)	14 (1–54)	13 (6–30)	6 (4–47)	0.63^ M^	0.70^ K^
Supraventricular tachyarrhythmia								
Number of participants	10 (10.3%)	2 (7.7%)	5 (23.8%)	2 (7.1%)	1 (4.5%)	2 (10.5%)	1.00^F^	0.22^F^
Events/24 h	3 (1–7)	2 (1–3)	2 (1–19)	7 (3–11)	1	2.5 (1–4)	0.69^ M^	0.31^ K^
PVC								
Number of participants	75 (77.3%)	18 (69.2%)	17 (81.0%)	23 (82.1%)	17 (77.3%)	10 (52.6%)	0.02^F^	0.64^F^
Events/24 h	35 (4–320)	3 (1–156)	44 (3–58)	74 (12–462)	35 (2–912)	1 (1–17)	0.17^ M^	0.03^ K^
PVC salvos								
Number of participants	14 (14.4%)	3 (11.5%)	2 (9.5%)	5 (17.9%)	4 (18.2%)	0 (0%)	0.12^F^	0.78^F^
Events/24 h	1 (1–5)	1 (1–2)	1 (1–1)	1 (1–5)	4 (1–8)	0	0.24^ M^	0.74^ K^
Ventricular tachyarrhythmia								
Number of participants	1 (1.0%)	0 (0%)	0 (0%)	1 (3.6%)	0 (0%)	0 (0%)	1.00^F^	1.00^F^
Events/24 h	1	0	0	1 (1–1)	0	0	0.32^ M^	0.45^ K^

Data are presented as total number (percentage) or median (interquartile range. * *p*-values refer to data comparison between the total patient group and controls. ** *p*-values refer to data comparison between all four ACHD subgroups. ^F^ = Fisher´s exact test; ^K^ = Kruskal–Wallis test; ^M^ = Mann–Whitney *U* test. *Abbreviations*: *PAC* premature atrial contraction, *PVC* premature ventricular contraction

HRV results are given in Table 4. Mean heart rate did not differ between patients and controls (71.0 ± 9.8/min vs. 68.1 ± 11.0/min, *p* = 0.24) nor over ACHD lesion groups (*p* = 0.66). In terms of frequency domain analysis, lnVLF (7.46 ± 0.76 ms^2^ vs. 7.91 ± 0.92ms^2^; *p* = 0.03), lnLF (6.39 ± 0.95ms^2^ vs. 7.01 ± 1.07ms^2^; *p* = 0.01) and lnLF/HF (0.81 ± 0.74 vs. 1.17 ± 0.51; *p* = 0.04) were significant lower in the patient than in the control cohort. With regard to time domain measures, no substantial differences were observed between patients and controls. When HRV measurements were examined over ACHD lesion groups, neither time nor frequency domain analysis showed significant differences in any of the studied parameters. Neither time nor frequency domain HRV parameters were significantly impacted by medication (in particular beta-blocker) or prior surgical intervention of the underlying cardiac defect. Correlation analysis revealed that NT-proBNP levels were significantly related to both time and frequency domain indices (lnSDNN: Spearman´s rho = -0.32, *p* = 0.001; lnSDANN: Spearman´s rho: -0.33, *p* = 0.001; lnTP: Spearman´s rho: -0.25, *p* = 0.01; lnVLF: Spearman´s rho: -0.33, *p* = 0.001; lnLF: Spearman´s rho: -0.35, *p* < 0.001; lnLF/HF: Spearman's rho: -0.34, *p* = 0.001).

**Table 4:** Heart rate variability measurements

	All patients (*n* = 97)	Shunt lesions (*n* = 26)	Left-sided obstructive lesions (*n* = 21)	Right-sided lesions (*n* = 28)	Complex lesions (*n* = 22)	Controls (*n* = 19)	*p*-value*	*p*-value**
Mean heart rate [beats/min]	71.0 ± 9.8	69.9 ± 9.2	73.4 ± 9.5	70.8 ± 9.4	70.4 ± 11.3	68.1 ± 11.0	0.24	0.66
Time domain analysis								
lnSDNN [ms]	4.90 ± 0.44	4.84 ± 0.32	4.95 ± 0.28	4.91 ± 0.68	4.90 ± 0.33	5.04 ± 0.34	0.20	0.86
lnSDANN [ms]	4.69 ± 0.41	4.71 ± 0.40	4.75 ± 0.55	4.57 ± 0.33	4.75 ± 0.34	4.89 ± 0.34	0.05	0.40
lnASDNN [ms]	4.05 ± 0.39	4.04 ± 0.37	4.05 ± 0.25	4.06 ± 0.37	4.05 ± 0.53	4.23 ± 0.48	0.83	1.00
lnRMSSD [ms]	3.75 ± 0.58	3.68 ± 0.53	3.67 ± 0.48	3.81 ± 0.66	3.83 ± 0.63	3.76 ± 0.59	0.94	0.70
lnPNN30 [%]	2.92 ± 0.83	2.92 ± 0.85	2.93 ± 0.74	2.89 ± 0.73	2.96 ± 1.04	3.23 ± 1.08	0.17	0.99
lnPNN50 [%]	2.25 ± 1.07	2.13 ± 1.08	2.19 ± 1.21	2.18 ± 0.99	2.53 ± 1.02	2.46 ± 1.48	0.48	0.58
Frequency domain analysis								
lnTP [ms^2^]	9.47 ± 13.97	7.96 ± 0.78	8.03 ± 0.50	13.23 ± 26.33	8.01 ± 1.03	8.41 ± 0.95	0.74	0.48
lnVLF [ms^2^]	7.46 ± 0.76	7.50 ± 0.74	7.51 ± 0.51	7.31 ± 0.78	7.53 ± 0.97	7.91 ± 0.92	0.03	0.73
lnLF [ms^2^]	6.39 ± 0.95	6.33 ± 0.86	6.52 ± 0.63	6.42 ± 0.80	6.28 ± 1.42	7.01 ± 1.07	0.01	0.86
lnHF [ms^2^]	5.59 ± 1.18	5.48 ± 1.23	5.57 ± 0.94	5.60 ± 1.25	5.74 ± 1.33	5.81 ± 1.28	0.49	0.91
lnLF/HF	0.81 ± 0.74	0.84 ± 0.77	0.95 ± 0.70	0.91 ± 0.83	0.53 ± 0.60	1.17 ± 0.51	0.04	0.26

Data are presented as mean ± standard deviation. * *p*-values refer to data comparison between the total patient group and controls (unpaired *t-*test). ** *p*-values refer to data comparison between all four ACHD subgroups (univariate ANOVA). *Abbreviations*: *ASDNN* average of the standard deviations of normal RR intervals for each 5-min segment, *HF* high frequency, *LF* low frequency, *LN* natural logarithm, *PNN30* percentage of RR intervals differing by more than 30 ms, *PNN50* percentage of RR intervals differing by more than 50 ms, *RMSSD* square root of the mean squared difference between normal RR intervals, *SDANN* standard deviation of the average normal RR intervals for each 5-min segment, *SDNN* standard deviation of all normal RR intervals, *TP* total power, *VLF* very low frequency

### Clinical outcomes

During a mean follow-up of 3.9 ± 0.7 years, 8 patients died. The causes of death comprised arrhythmia (*n* = 1), SCD (*n* = 4) and heart failure (*n* = 3). SCD due to ventricular fibrillation occurred in 3 patients who were successfully resuscitated and thereafter underwent implantation of implantable cardioverter-defibrillator for secondary prevention. Almost one in three ACHD patients were hospitalized for at least one time during follow-up (*n* = 31, 32.0%). 27 (27.8%) patients had at least one event of acute cardiac decompensation during follow-up. These events comprised acute decompensated heart failure due to fluid retention requiring medical therapy optimization that was not necessarily accompanied by hospital admission. On Cox regression analysis, only the time domain indices lnSDANN and lnRMSSD were associated with acute cardiac decompensation. With regard to hospital admission, only lnSDANN showed an association.

To analyse the impact of HRV parameters on patients’ event-free survival, a survival analysis by using a univariate Cox regression analysis was conducted. Although the group of deceased ACHD patients and SCD survivors had higher NT-proBNP levels (333 pg/ml (IQR 90–2492) vs. 135 pg/ml (IQR 54–316)) and decreased EF (48.0 ± 12.4% vs. 58.9 ± 8.11%), these differences were not statistically significant (Table 5).

**Table 5:** Demographic and clinical features of ACHD event-free survivors (censored) and those who died or survived SCD

	Censored event-free survivors	Deceased or survived SCD patients during examination interval	Hazard ratio (95% CI)	*p*-value
Demographics				
Female	46 (52.3%)	4 (44.4%)	0.82 (0.22–3.12)	0.78
Age [years]	39.4 ± 14.4	36.6 ± 11.1	0.98 (0.93–1.03)	0.49
Previous reparative intervention	69 (78.4%)	8 (88.9%)	2.26 (0.28–18.07)	0.44
NYHA functional class			1.53 (0.73–3.20)	0.26
I	50 (56.8%)	2 (22.2%)		
II	32 (36.4%)	6 (66.7%)		
III	6 (6.8%)	0 (0.0%)		
IV	0 (0%)	1 (11.1%)		
Biochemistry				
NT-proBNP [pg/ml]	135 (54–316)	333 (90–2492)	1 (1.00–1.00)	0.20
NT-proBNP ≥ 125 [pg/ml]	42 (47.7%)	6 (66.7%)	1.23 (0.31–5.20)	0.74
NT-pro BNP ≥ 300 [pg/ml]	23 (26.1%)	5 (55.6%)	2.31 (0.61–8.75)	0.22
Systemic ventricle EF [%]	58.9 ± 8.11	48.0 ± 12.4	0.95 (0.89–1.01)	0.09
ACHD lesion group			1.63 (0.83–3.22)	0.16
Shunt lesions	25 (28.4%)	1 (11.1%)		
Left-sided obstructive lesions	21 (23.9%)	0 (0%)		
Right-sided lesions	23 (26.1%)	5 (55.6%)		
Complex lesions	19 (21.6%)	3 (33.3%)		

Data are presented as total number (percentage), mean ± standard deviation or median (interquartile range). *Abbreviations*: *ACHD* adult congenital heart disease, *CI* confidence interval, *EF* ejection fraction, *NT-proBNP* N-terminal prohormone of brain natriuretic peptide, *NYHA* New York Heart Association

**Fig. 1:**
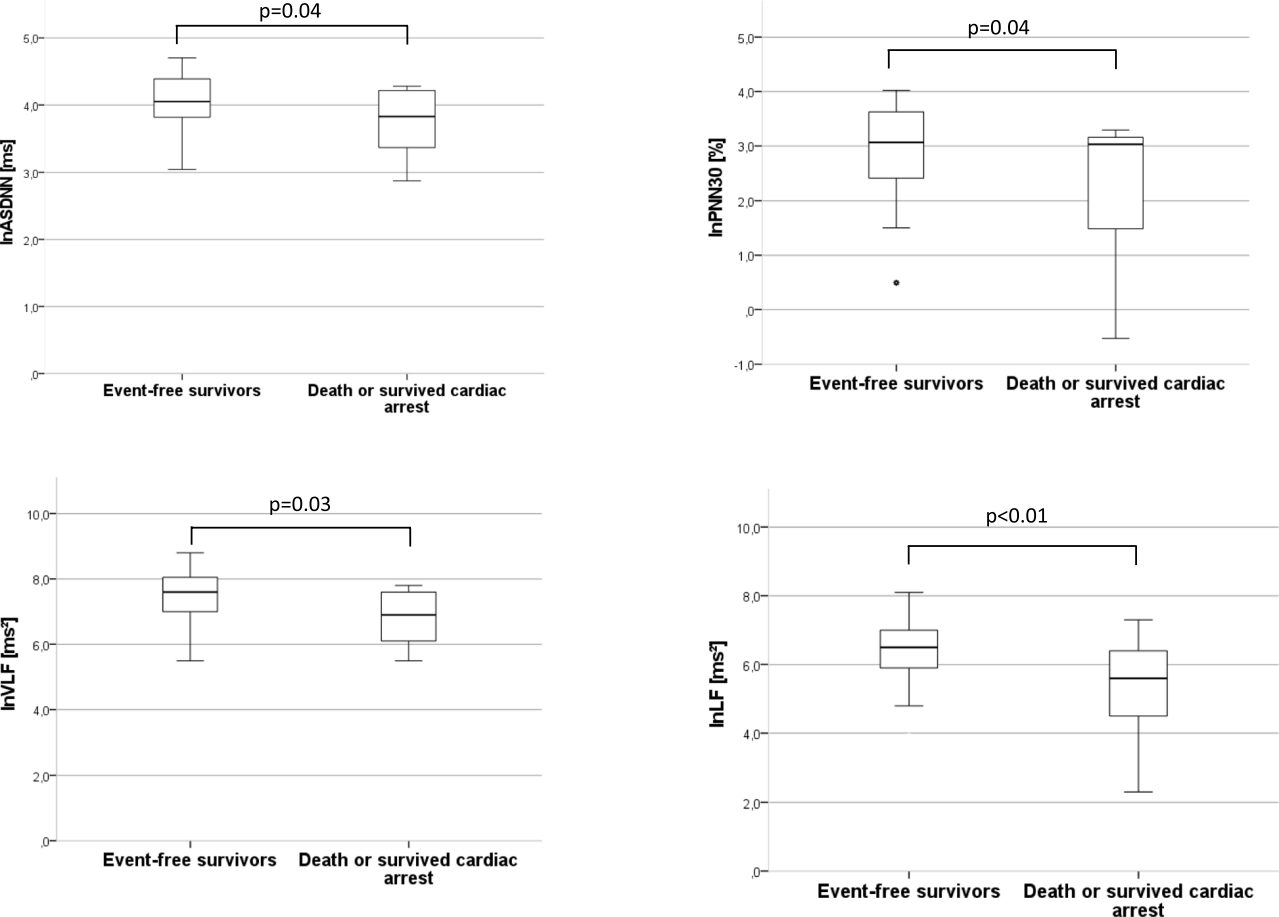
HRV measurements in event-free ACHD survivors (censored) and patients who died or survived cardiac arrest during follow-up. Abbreviations: ASDNN: average of the standard deviations of normal RR intervals for each 5-min segment; LF: low frequency; LN: natural logarithm; PNN30: percentage of RR intervals differing by more than 30 ms; VLF: very low frequency

In terms of HRV measurements, when HRV measurements were compared between event-free survivors and those who died or survived SCD, decreases in HRV were observed in the patient group that comprised deceased and SCD survivors (lnASDNN: 3.73 ± 0.55 ms vs. 4.07 ± 0.36 ms, *p* = 0.04; lnPNN30: 2.25 ± 1.51% vs. 2.97 ± 0.75%, *p* = 0.04; lnVFL: 6.80 ± 0.89 ms^2^ vs. 7.50 ± 0.74 ms^2^, *p* = 0.03; lnLF: 5.28 ± 1.76 ms^2^ vs. 6.47 ± 0.83 ms^2^, *p* < 0.01; Fig. 1).

On univariate Cox regression analysis, the time domain indices lnSDNN, lnASDNN and lnPNN30, as well as the frequency domain parameters lnTP, lnVLF and lnLF were associated with death or survived SCD (Table 6). In due consideration of the low event rate (*n* = 11), no additional multivariate Cox proportional hazard regression analysis was performed [12].

**Table 6:** Univariate Cox regression analysis

	Hazard ratio (95% CI)	*P*-value
lnSDNN	0.024 (0.001–0.624)	**0.03**
lnSDANN	0.273 (0.053–1.397)	0.12
lnASDNN	0.104 (0.012–0.871)	**0.04**
lnRMSSD	0.285 (0.049–1.671)	0.16
lnPNN30	0.458 (0.224–0,936)	**0.03**
lnPNN50	1.146 (0.487–2,698)	0.76
lnTP	0.280 (0.95–0,829)	**0.02**
lnVLF	0.289 (0,093–0,903)	**0.03**
lnLF	0.432 (0.0247–0,756)	** < 0.01**
lnHF	0.603 (0.279–1,303)	0.20
lnLF/HF	0.464 (0.162–1.327)	0.15

Statistically significant differences are given in bold. *Abbreviations*: *ASDNN* average of the standard deviations of normal RR intervals for each 5-min segment, *CI* confidence interval, *HF* high frequency, *LF* low frequency, *LN* natural logarithm, *PNN30* percentage of RR intervals differing by more than 30 ms, *PNN50* percentage of RR intervals differing by more than 50 ms, *RMSSD* square root of the mean squared difference between normal RR intervals, *SDANN* standard deviation of the average normal RR intervals for each 5-min segment, *SDNN* standard deviation of all normal RR intervals, *TP* total power, *VLF* very low frequency

## Discussion

The present study prospectively analysed HRV alterations and their association with outcome in ACHD patients. The main results are as follows:

1. When compared to healthy controls, ACHD patients exhibited an impairment in HRV as assessed by time and frequency domain analysis.
2. HRV measurements did not differ over ACHD lesion groups.
3. Both frequency and time domain indices carried prognostic implication on ACHD mortality and survived cardiac arrest.

Arrhythmias are a leading cause of hospitalization and cardiac death in the ACHD population [13, 14]. The aetiologies from which ACHD patients are subject to rhythm disorders comprise arrhythmic sequelae associated with the specific underlying heart defect, conduction abnormalities, surgical scar, hypoxia and abnormal pressure/volume loads of long duration [15]. As a consequence of the high incidence and prognostic implication of arrhythmias, early diagnosis is desirable. In general terms, the autonomic nervous system plays an important role in the initiation and sustaining of arrhythmias. It is the prime regulator of heart rate and determines HRV [6]. HRV, i.e., the amount of heart rate fluctuation around the mean heart rate, can be quantified by different methods of which time and frequency domain analysis represents a non-invasive approach of good reproducibility. The traditional applications of HRV measurement are the surveillance of post-myocardial infarction and diabetic patients. Moreover, decreased HRV has also been shown to be present and to serve as a strong and independent predictor of adverse outcome in patients with stable coronary heart disease, heart failure, atrial fibrillation and pulmonary hypertension [16, 17]. The value of HRV analysis in the field of ACHD has been subject of recent research. These studies have principally focused on single defects and the impact of previous surgical repair on HRV. In an ACHD cohort of 30 surgically closed and 30 unrepaired ventricular septal defects that underwent time domain analysis, Maagard et al. ascertained an impaired HRV and higher proportion of PVC in ACHD patients when compared to their healthy peers. Differences were highest amongst patients with surgically closed ventricular septal defects [18]. Caution should be made in direct comparison of Maagard´s results to our observations as HRV indices did not undergo logarithmic transformation. Moreover, we additionally measured frequency domain indices. Except for HF and total power, frequency domain measurements were significantly impaired in ACHD patients, irrespective of the underlying ACHD lesion group. In light of our findings, it is worth considering briefly the separate rhythmic contributions of sympathetic and parasympathetic autonomic activity. While sympathetic activity modulates heart rate in the low frequency range (0.04–0.15 Hz), parasympathetic activity is related to the high frequency range (0.15–0.40 Hz) [6, 19]. In keeping with this and the currently observed unaffected HF spectrum among ACHD patients, our results hint at an autonomic dysfunction that primarily affects the sympathetic branch, whilst the parasympathetic tone is mainly preserved.

With regard to the prognostic value of HRV measurements, several time and frequency domain indices were presently identified to be associated with mortality in ACHD on univariate regression analysis. Taking into account the recommended number of 10 events per independent variable in proportional hazards regression analyses [12] and the currently observed event rate of 8 deaths, no multivariate analysis was performed. Decreases in SDNN and VFL power have been reported to predict outcome in patients with acute myocardial infarction [20]. In stable angina pectoris, alterations in frequency domain measurements have been shown to independently predict cardiovascular death [21]. In the UK-HEART study, the HRV measurement SDNN was the most powerful predictor of mortality due to progressive heart failure [22]. However, HRV analysis was confined to time domain measurement, frequency domain indices were not assessed. Within the field of ACHD, Lammers et al. examined HRV by time domain analysis and additionally assessed heart rate turbulence in 43 ACHD patients [5]. They identified pathologic heart rate turbulence to be the only independent predictor of all-cause mortality, whilst time domain measures had no predictive value. These results are only partially comparable with our observations. Consistent with Lammers and colleagues, we ascertained the time domain index SDNN to be associated with mortality. Contrary to them, we additionally performed frequency domain analysis and hereby obtained a wide range of parameters that were related to death. However, the question whether theses indices independently carry prognostic implication could not be answered definitively in due consideration of the limited number of deaths observed.

There are several limitations that should be addressed. First, we assume that selection and procedure bias might have arisen from our single-centre study design. This single-site character impeded the enrolment of a larger number of controls and ACHD patients with higher event rates and the option to perform multivariate hazard regression analysis. Secondly, we subdivided ACHD patients according to their haemodynamics to assess intergroup differences. It adheres to current guidelines, but might entail the risk of disregarding other, more appropriate discriminants. Finally, our ACHD patient cohort encompassed a broad spectrum of congenital heart lesions within which some defects were underrepresented. It thereby restricts generalizability of our study results.

In conclusion, ACHD is accompanied by HRV impairment. Altered time and frequency domain indices carry prognostic information. Our results set the stage for larger trials with higher patient numbers and longer duration of follow-up to confirm the predictive value of HRV measurements and deduce preventive approaches.

## Data Availability

The datasets generated during and/or analysed during the current study are available from the corresponding author on reasonable request.
